# Towards a Research Programme Aiming at Causes and Consequences of Reticulate Evolution

**DOI:** 10.3390/biology14111601

**Published:** 2025-11-15

**Authors:** Christoph Oberprieler

**Affiliations:** Evolutionary and Systematic Botany Group, Institute of Plant Sciences, University of Regensburg, Universitätsstr. 31, D-95053 Regensburg, Germany; christoph.oberprieler@ur.de; Tel.: +49-941-943-3129

**Keywords:** biological complexity, biological diversity, endosymbiosis, evolving systems, hybridisation, hybrid speciation, organisms, phylogenetics, symbiosis, systematics, theory of evolution

## Abstract

Evolution is reticulate. Reticulation increases diversity and complexity on the different levels of the evolutionary hierarchy. In addition to the tendency for diversity and complexity to increase in unchecked evolutionary systems by ongoing divergence (‘Zero-Force Evolutionary Law’, ‘Biology’s First Law’), reticulate evolution, therefore, acts as a second mechanism for the establishment of evolutionary novelty and the rise in biodiversity and biocomplexity (‘Biology’s Second Law’). This provides the raw material for subsequent diversity-confining drift and selection processes. In order to fully appreciate reticulation processes as part of an updated paradigm of evolutionary biology, a research programme on the topic should encompass the identification of the fundamental evolutionary entities as vertices and the study of the relationships among these vertices as edges in the resulting network architectures. Additionally, along with surveys on the underlying determinants, this will lead to the study of emergent boundary conditions for reticulations and for the porosity of evolutionary entities. Finally, the programme should address the question whether there are equilibrium conditions between the complete fusion and complete isolation of evolutionary entities (‘Goldilocks Zones’) that foster reticulate evolution. As tools in this research programme, machine learning and modelling approaches, along with methods in the field of network reconstruction, transcriptomics, epigenetics, and karyology, are identified.

## Simple Summary

Modern evolutionary theory still rests on a ‘Tree of Life’ (ToL) metaphor. It explains the origin and growth of diversity and complexity in biological systems mostly by processes of ongoing divergence among its units when unchecked by evolutionary forces like selection or genetic drift (‘Zero-Force Evolutionary Law’). This view of divergence as the default process in biological diversification has been considered as being ‘Biology’s First Law’, which could be applied to all levels of the biological hierarchy (genes, genomes, cells, tissues, organisms, populations, species, clades). The present review tries to draw attention to evolutionary processes that lead to the origin and growth of diversity and complexity in biological systems by reticulations (endosymbiosis, symbiosis, hybridisation, hybrid speciation) and argues for attributing to these processes an equally fundamental significance in evolutionary biology (‘Biology’s Second Law’). Additionally, a theoretical framework is provided for generalising among the resulting patterns of evolutionary reticulations across all mentioned levels of the biological hierarchy. These may reach from a complete merger of evolutionary entities at a certain level, over the exchange of lower-level units among these entities, to the formation of novel evolutionary entities. Finally, the present contribution provides a comprehensive list of examples for reticulate evolutionary processes at the different levels of the biological hierarchy and their potential outcome and makes suggestions for scientific research questions and tools to further increase the appreciation of reticulation processes in evolutionary theory.

## 1. Introduction

Evolutionary biology plays a crucial role in biodiversity research by providing the framework and tools for understanding the origins, patterns, and maintenance of biological diversity. In contrast to ecological approaches, evolution-focussed biodiversity research usually works on larger temporal and spatial scales and contributes to the above-mentioned general aims by exploring the origin of evolutionary significant entities (species), studying the moulding of these entities by evolutionary processes like adaptation, resolving the evolutionary relationships among species (phylogenetics), explaining the spatial and temporal patterns of diversity and responses to environmental change (biogeography), informing taxonomy as a tool for exchanging information about biodiversity, and, as a consequence, forming a reference system for biodiversity changes and allowing suggestions for its conservation.

During the last 25 years, the conceptual framework of the ‘Modern Synthesis’ (MS) of evolutionary biology formulated in the 1940s was considered to be in need of considerable extensions due to the integration of findings in the fields of ‘molecular biology and evolutionary developmental biology, the recognition of ecological development, niche reconstruction and multiple inheritance systems, the “-omics” revolution and the science of systems biology’ ([1], see also [2,3,4]). While the resulting Extended Evolutionary Synthesis (EES) acknowledges the incorporation of novel findings concerning the origin, innovation, and inheritance of traits and genome evolution (epigenetics, mobile elements, repetitive elements, non-coding RNAs), Evo-devo theory, or the concepts of phenotypic plasticity and niche construction, aspects of reticulate evolution are just beginning to find reverberation as additional and extremely important contributions to this new conceptual edifice of the biological sciences.

Whole-genome sequencing projects in the last decade provided convincing support for the fact that the ‘Tree of Life’ (ToL) metaphor—the perception of organismic evolution as an ever-diverging, novel lineage producing process (‘Biology’s First Law’; [5,6]; ‘Tree thinking’; [7])—is an oversimplification not only in the prokaryotic and plant kingdoms, but throughout the eukaryotic world [8,9,10,11]. Therefore, the ubiquity and innovative potential of reticulate evolutionary processes (symbiosis, symbiogenesis, lateral DNA transfer, hybridisation, infectious heredity; [12]) are considered to be undeniable arguments for the inclusion of these processes into an updated paradigm of evolutionary biology [13]. This effort is presently paralleled by paradigmatic shifts in the non-biological natural sciences (physics, cosmology, chemistry, geology, etc.) aiming to find the underlying laws for evolving systems in general (‘Assembly theory’, [14], but see [15]; ‘Law of increasing functional information’, [16], but see [17]).

The aims of the present contribution are, therefore, to give a brief summary of the state of the art in the research field of evolutionary biology dealing with the phenomenon of reticulate evolution (phenomenological approach to reticulate evolution), to find general patterns and fundamental processes of reticulate evolution across the different levels of evolutionary entities (theoretical approach to reticulate evolution from the perspective of Tëmkin and Eldredge’s [18] ‘Hierarchy Theory of Evolution’, but see [19]), to tag determinants and boundary conditions shaping the trajectories of reticulations, and to identify future directions of research programmes aiming at the causes and consequences of reticulate evolution in order to fully address the importance of evolutionary reticulations in an updated paradigm of evolutionary biology.

## 2. Levels of Reticulate Evolution—The State of the Art

### 2.1. Ultra-Deep Reticulations—Symbiosis, Endosymbiosis, and Inter-Clade Horizontal DNA Transfer

Despite the high number of competing hypotheses concerning the origin of the eukaryotic cell [20], there is no doubt about the extreme importance of the subsequent engulfments of additional prokaryotic and eukaryotic cells (endosymbiosis) for major transitions in the evolution of eukaryotes. Recent phylogenomic analyses aiming at the eukaryote Tree of Life (eToL) have revealed many novel lineages and relationships, especially in the group of microbial ‘protists’ [21,22,23]. While the serial endosymbiosis events in the evolution of (micro)algae, with their connected DNA transfers from endosymbionts to the host genome, are a well-established theory, the sequence, timing, and involved partners of these events remain uncertain [24]. Additionally, the factors influencing the transition of engulfed microorganisms from prey to elementary and obligate constituents of the predatory host cell and the loss or exchange of endosymbiotic plastids (via ‘kleptochloroplasts’), especially in mixotrophic lineages, are a matter of ongoing research [25,26]. Owing to the fact that mixotrophic plankton are expected to become more prevalent in future ocean habitats [24,27], the boundary conditions determining prerequisites and outcomes of evolutionary reticulations caused by endosymbiosis will be increasingly important topics of research in the ‘protist’ realm.

Besides eubacterial endosymbiotic associations that evolved in the distant ancestors of modern eukaryotes, the symbiosis of eukaryotes with bacterial and eukaryotic microorganisms are ‘a general principle in eukaryotic evolution’ [28]. These symbiotic associations may provide multicellular eukaryotes with adaptive and evolutionary stimuli by enabling novel lifestyles through the gaining of new metabolic traits (photosynthesis in lichens, nitrogen fixation in numerous plant groups, carbon dioxide fixation in animals living in aquatic sediments, hydrothermal vents, and hydrocarbon seeps, or lignocellulose digestion in insect guts) or causing changes in their reproductive systems (e.g., incompatibility due to *Wolbachia* infections in arthropods). Additionally, advances in environmental DNA (eDNA) sampling have shown that ‘the vast majority of organisms live intimately with many other species from all kingdoms of life’ that do not necessarily involve cell mergers [29]. The resulting microbiome of a eukaryotic organism may therefore have significant developmental and ecological influences and may show all stages and transitions among all possible types of symbiosis (neutralism, commensalism, amensalism, mutualism, parasitism, and synnecrosis). As a consequence, the ‘holobiont’ (i.e., the multicellular plant, fungi, or animal individual together with its associated microbiome) may be viewed as a complex evolving entity [29,30,31].

The formation of a multi-taxonomic holobiont opens ample possibilities for horizontal gene/DNA transfer (HGT) via the incomplete digestion of engulfed food cells, extracellular transport vesicles (microvesicles, membrane vesicles, micro- and nanoparticles, ecto- and exosomes), or the activity of viruses, virus-like particles, and giant viruses [29]. Despite the observation that ‘the magnitude of HGT in eukaryotes is markedly inferior to that in bacteria and archaea’ [32], the growing numbers of high-quality genome data from eukaryotes reveal the significant contribution of this process to the architecture of eukaryotic genomes and its role in adaptive changes in evolutionary lineages [33]. As a consequence, there are copious examples of inter-phylum adaptive horizontal DNA transfers [34] (p. 12f); [29] (p. 22, footnote f), including, among others, such impressive cases as enzymes for cellulose digestion from bacteria or fungi to plant-pathogenic nematodes [35,36], anti-fungal defence proteins from a beetle to a nematode [37], bacterial and fungal genes into human-pathogen fungi [38], and HGT among plants [39] or in insects [40]. Holobionts, therefore, may be viewed upon as having collective genomes (‘hologenomes’, [30]) that form—together with transport vectors like vesicles, vectors, and viruses—‘evolutionary melting pots’, allowing for fast reactions to ecological transitions by triggering genomic changes via genomic reticulations.

### 2.2. Deep Reticulations—Reticulate Phylogenetics

Due to the considerably long time scales involved, the study of evolutionary processes—like speciation and differentiation, but also reticulation processes—in groups of closely related, non-model eukaryotic lineages is mostly performed non-experimentally by the application of either of two approaches: a magnifying glass or a spyglass approach [41]. While the former addresses the mentioned processes by zooming into populations and monitoring short-term (micro)evolutionary changes (see scientific focus 3 under point 2.3), the latter applies phylogenetic reconstructions to infer the circumstances and the impact of these processes by looking back into the long-lasting (macro)evolutionary history of an organism group. The reconstruction of ancient hybridisation events leading to deep reticulations in the corresponding phylogenies has been improved tremendously by the advances in sequencing technologies allowing the analysis of whole genomes at low costs. The simultaneous development of analytical pipelines for the inference of phylogenetic networks has further enabled scientists to study the long legacy of hybridisation and its macroevolutionary significance in eukaryotic organisms.

Whole-genome sequencing (genomics) and the phylogenetic analysis of these data (phylogenomics) over the last decade have revealed how reticulate the evolutionary history of animals [42] and plants [43] has been. Ancient hybridisation, partial- and whole-genome duplication, and lateral gene/DNA transfer processes have left their long-lasting fingerprint in eukaryotic genomes. Therefore, deep reticulations caused by ancient hybridisation events are assumed for numerous families, orders, or even larger clades (Rosids, Asterids) in the plant kingdom [43]. While reticulation events caused by relatively recent allopolyploidy (WGD) could be pinpointed to branches in a phylogeny and to certain geological periods with present phylogenomic tools (e.g., in Caryophyllaceae by [44]), inferring discrete hybridisation (without genome duplications) and HGT events is beyond the abilities of current methodology due to the erosion of a hybrid signal over time or the extinction of parental lineages [43]. Additionally, repeated cycles of deep reticulations caused by (allo)polyploid whole-genome duplications and followed by diploidisation processes through translocations, chromosome fusions, and other changes in chromosome architecture (‘genome tectonics’, [45]) have further contributed to difficulties in phylogenomic reconstructions, especially gene-tree incongruences and consequently species-tree uncertainties.

As a solution to these problems, and especially to address the disentanglement of ancient hybridisation from confounding effects of incomplete lineage-sorting (ILS), coalescent-based species-network inference methods (PhyloNetworks, [46]; PhyloNet, [47]; HyDe, [48]) are used. More recently, synteny-based analyses of highly contiguous genome assemblies are suggested and were successfully applied in deep, recalcitrant phylogenetic problems like metazoan and pre-metazoan evolution [45,49] or relationships among major groups of teleost fishes [50]. However, reticulate evolution may even in these synteny-based methods provide a source of phylogenomic error or noise [51]. Additionally, the combination of sequence-based phylogenetic analyses with cytogenetic and cytogenomic techniques that are capable of capturing rearrangements of chromosomal architecture during diploidisation processes [fluorescent in situ hybridisation (FISH), genome in situ hybridisation (GISH), or chromosome painting (CP); [52]] will further help to infer ancient reticulate evolutionary events.

Accepting that the Tree of Life metaphor is unrealistic and overly simplistic for both prokaryote and eukaryote evolution, it should be replaced by a Net of Life perspective. Many current tree-based methods for reconstructing trait evolution, as well as the temporal, spatial, geological, and eco-climatological aspects of organismal evolution (e.g., biogeography, niche evolution), will need reconsideration and redesign [43]. With these new tools, we can better account for the macroevolutionary importance of hybridisation and gain deeper insights into reticulate processes in organismal diversification. Additionally, we can test whether punctuated equilibria in the evolutionary history of some groups can be explained by shifts in reticulation frequency driven by geological or paleoclimatological changes. Retrospective analyses of the conditions enabling hybridisation, combined with comparisons to recent reticulation events (see scientific focus 3 under point 2.3), will also support predictions about the role of hybridisation in shaping biodiversity under future environmental changes.

### 2.3. Shallow Reticulations—Hybridisation and Homoploid or Polyploid Hybrid Speciation

It is hardly possible to overestimate the importance of hybridisation in plant evolution and to elude the argumentation of Oberprieler [53] that *Biology’s First Law* [5], saying that ‘in the absence of selection and constraint, complexity—in the sense of differentiation among parts—will tend to increase’, should be augmented by a second principle (and maybe *Biology’s Second Law*) that complexity does not only increase through differentiation and divergence alone but also through genetic exchange, (re)combination, and phylogenetic reticulation. We are presently experiencing a shift in perspective from the view of hybridisation as a merely destructive process that could lead to a reversal of differentiation and a loss of biodiversity, towards an enforced appreciation of hybridisation as a constructive and even creative process [8,9,10,11,54,55,56]. Human-mediated hybridisation events caused by land-use changes, the introduction of non-native species, and rapid climate change may support the view of this process being a paramount signature of the Anthropocene [57,58]. From an evolutionary perspective, the study of these presently occurring shallow reticulations offers opportunities for understanding the causes, boundary conditions, and consequences of this significant process that are not easily inferable from deep reticulation reconstructions (see scientific focus 2 under point 2.2).

The genetic and genomic consequences of hybridisation are numerous and range from alterations of epigenetic modifications and expression patterns across the genome, over the activation and spread of mobile DNA elements with their genome- and karyotype-reconstructuring ramifications, to adaptive horizontal DNA/gene transfer via the introgression and the formation of new species via homoploid and polyploid hybrid speciation [34,59]. With an estimated frequency of at least 25% of species that hybridise with each other [60], the plant kingdom represents a well-suited domain of life for studying this paramount evolutionary process. However, the more-or-less pervasive nature of hybridisation processes is also documented in all other large groups of eukaryotes (protozoa, [61]; fungi, [62]; metazoa, [63]).

Being two sides of a coin, research into the causes and consequences of hybridisation is tightly connected with speciation research [54,64]. This is not only due to the mentioned hybrid speciation processes, but also because the analysis of ‘porous genomes’ allows the elucidation of the genetic and genomic background involved in the evolution of reproductive barriers, and hybridisation often features as the final step in the formation of prezygotically isolated ‘biological species’ through a so-called reinforcement mechanism [65]. Additionally, cataclysmic genetic and epigenetic changes caused by hybridisation events (‘hybridisation shock’) also create the necessary genomic innovations allowing environmental adaptations, morphological and physiological diversifications, and adaptive radiations (e.g., in birds, [66], or in cichlid fishes, [67]). In other groups (e.g., in tropical eels, [68]) it has been shown that, despite pervasive hybridisation, species boundaries have been stable throughout millions of years.

Besides the empirical work on speciation/hybridisation across all eukaryote parts of the Tree of Life throughout the last decades, and especially propelled by progresses in molecular biology (whole-genome sequencing, analysis of ncRNAs, transposable elements, repetitive elements, epigenetic conditions, CRISPR/Cas techniques), the evolutionary significance of reticulations has also found its consideration in theoretical biology. The mathematical modelling of hybridisation [69] and of the build-up of reproductive isolation by multi-locus interactions [70], along with the search for the boundary conditions involved in hybrid speciation [56], may allow predictions about the role and interplay of the extrinsic (environmental) and intrinsic (genetic, epigenetic, chromosome architectural; [71]) circumstances giving rise to hybridisation, controlling its frequency, and shaping its evolutionary outcomes and its contribution to organismal diversity.

## 3. Generalisations and Specifications

### 3.1. Evolutionary Entities—Levels of Units Showing Heritable Variation

Biological organisation spans multiple levels, from the molecular to the ecological and phylogenetically one: genes, genomes, organelles, cells, tissues, organs, individuals, populations, species, and clades or ecosystems. Each level emerges through interactions at the preceding level, forming a structured system in which selection and other evolutionary forces can operate. This view of the hierarchical organisation of evolutionary entities is central to McShea and Brandon’s [5] (p. 9) definition of *diversity* and *complexity* as the ‘number of part types or differentiation among parts’ at a lower level enclosed in the adjacent upper level. This allows the authors to formulate their ‘Zero-Force Evolutionary Law’ (ZFEL) predicting that, in the absence of natural selection, other forces, and constraints, diversity and complexity will increase on average in any evolutionary system (i.e., on every focal level in the hierarchy of evolutionary entities).

In McShea and Brandon’s [5] terminology, the *diversity* of an evolutionary unit is defined by the number of different (lower-level) kinds or units it comprises, while the *complexity* (in their words ‘*pure complexity*’) of an evolutionary unit takes into account the differentiation among these (lower-level) kinds or units. It should be mentioned here that alternative concepts of *diversity* and *complexity* exist that, for example, (a) take into account the proportional abundance of (lower-level) kinds or units—as measured, for example, by Shannon’s diversity index—together with the divergence among these (lower-level) kinds or units when describing the *diversity* of an evolutionary unit, and (b) regard as an additional aspect of the *complexity* of an evolutionary unit (or network) the number and the nature of the interdependent relationships (network edges) among its lower-level kinds or units (network vertices). In the following, I will stick to McShea and Brandon’s [5] quite narrow definitions of the two terms in order to keep confusion at a minimum.

According to Tëmkin and Eldredge’s [18] ‘Hierarchy Theory of Evolution’, this multi-level arrangement of entities forms genealogically and ecologically nested compositional hierarchies, in which the ‘interactions of entities at a lower level establish *boundary conditions* (*upward causation*) and interactions of entities at a higher level exert constraints, or determine *initiating conditions* (*downward causation*)’ [72]. Tëmkin and Eldredge’s [18] theory appears also to be helpful in solving the long-lasting and controversial ‘units and levels of selection’ discussion [73] and paving the way towards the appreciation of multi-level selection [74], in allowing for a consistent perspective of the genesis of emergent and innovative characteristics in biological systems as being the upward consequences of lower-level differentiations [75] and in explaining the impression of teleology in evolution as being the result of the observed behaviour of focal entities on a lower level directed by a *field* (gradients, biases, boundaries, constraints, or contexts) spawned by the adjacent upper level [76].

Without going into an extensive discussion in philosophical respects, it should be mentioned here that the ‘Hierarchy Theory of Evolution’ as described by Tëmkin and Eldredge [18] also received criticism, especially in terms of its assumptions concerning the interaction between its genealogical and economical/ecological hierarchies. Gontier [19] argues that the model of upward and downward causations, limited to their independent agency along the two columns or strands of single ontological hierarchies, is incomplete and that research on macroevolution and reticulate evolution brings forth the need for interactional hierarchies and the introduction of ‘reticulate causation’ (see also [77,78]). The integration of holobiont formation at lower and geobiome formation at higher levels of the interacting genealogical (‘Linnaean’) and economical (‘Vernadskyan’) hierarchies led Spiridonov and Eldredge [79] to the proposal of a hybrid eco-genealogical (‘Bretskyan’) hierarchy that still shows a nestedness of entities and lacks the inclusion of Gontier’s [19] reticulate causation. It is beyond the scope of the present contribution to solve these philosophical controversies; however, Gontier’s [19] ‘Applied Evolutionary Epistemological (AEE)’ approach may at least help empirically working evolutionary biologists to clarify what is a *unit* of evolution, what is a *level* of evolution, and what is a *mechanism* or a *process* of evolution.

### 3.2. What Is Evolutionary Divergence?—Biology’s First Law

As mentioned above, McShea and Brandon’s [5] (p. 9) ‘Zero-Force Evolutionary Law’ (ZFEL) applies at ‘all levels where there is heritable variation’ and ‘predicts increase in diversity and complexity of genes, macromolecules, organelles, cells, tissues, organs, individuals, groups, populations, species, clades, or higher-level units.’ Thinking in terms of Tëmkin and Eldredge’s [18] ‘Hierarchy Theory of Evolution’, in which entities of evolution at each hierarchical level show differences and interactions among each other, ongoing differentiation among these entities increases the diversity and complexity of the next-higher-level entity encompassing these lower-level units. For instance, as the diversity of a tissue increases with the growing number of individual cell types involved, its complexity increases with the growing differentiation among these cell types. Or the diversity of a species increases with the number of divergent populations it comprises, while its complexity is determined by the amount of divergence among populations in this metapopulation system (as measured, for example, by *F_ST_* values).

The divergence among entities at a given focal level of the evolutionary hierarchy results from the interruption of interactions among these entities, leading to a downward-directed rupture of interactions among entities at lower hierarchical levels through to the lowermost level (genes), paralleled by the ongoing diversification among the now isolated, independently evolving, hierarchical entities at the focal level. For example, in a speciation process, interaction among contained lower-level entities (populations) of the focal entity (species) are interrupted, leading to the decomposition of entities at all lower hierarchical levels (down to the now orthologs of a gene) and the independent evolution of these two newly established systems of hierarchical levels. Additionally, this process leads also to the further growth of complexity in the adjacently higher level (clade) to the focal one.

### 3.3. What Results from Evolutionary Reticulation?—Biology’s Second Law

Evolutionary reticulations, as illustrated by the many examples in the first part of the present contribution, represent processes opposite to evolutionary divergence and may have three different outcomes (‘evolutionary arithmetics’): (a) the complete merging of two evolutionary entities (1 + 1 = 1), (b) the exchange of lower-level evolutionary entities between entities at the focal level (1 + 1 = 2), and (c) the emergence of an additional evolutionary entity at the focal level (1 + 1 = 3). In parallel to the different names of entities at different levels in the hierarchy of evolutionary units (genes, genomes, organelles, populations, species, etc.) and the different outcomes described, reticulate processes have received different terms in evolutionary biology (e.g., recombination, endosymbiosis, symbiosis, gene flow, hybridisation, horizontal DNA transfer, introgression, hybrid speciation, etc.).

**Figure 1:**
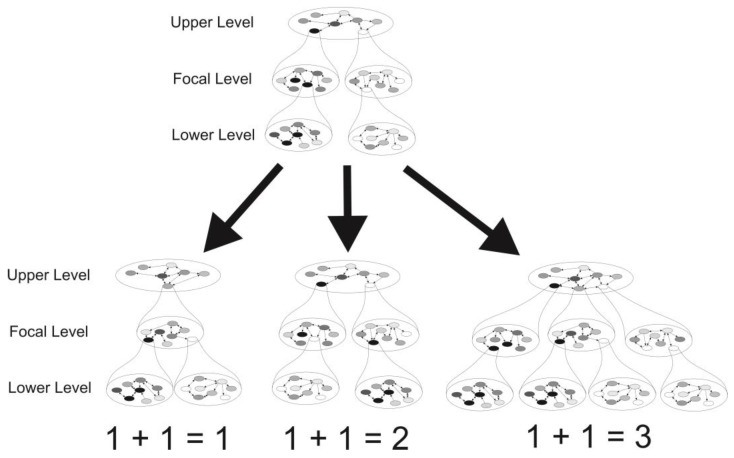
The architecture of a nested compositional hierarchy of biological entities and the potential outcomes of reticulation events among entities at a focal level. Intra-level interactions (arrows) connect individual entities (circles) within networks at each level. Three possible results of a collision of two entities at the focal level and their consequences for the composition of entities and networks at the upper- and lower-next level are depicted: scenario (1 + 1 = 1) describes the complete merger of the two entities at the focal level, while scenario (1 + 1 = 2) presents an exchange of lower-level entities between those two. Finally, scenario (1 + 1 = 3) illustrates an expected outcome in the case of a reticulate formation of a novel entity at the focal level.

When applying McShea and Brandon’s [5] definitions of the *complexity* of an entity at a focal level as the ‘number of part types or differentiation among parts’ (i.e., the diversity of entities at the adjacent lower level) and the *diversity* of entities at a given level as the number of entities and differentiation among them (i.e., the complexity of the containing entity at the adjacent higher level), we can make the following predictions for the three different reticulation scenarios described above (see Figure 1 for illustration):

In scenario (1 + 1 = 1), the merging of two entities at the focal level will increase the average diversity and average complexity at this level (relative to the average diversity and complexity levels before the merger), but will lead to a decrease in the diversity and complexity of the adjacent higher level, while the average diversity and complexity at the adjacent lower level will remain constant. Finally, the trajectories of diversity and complexity at all subsequent lower levels will depend on the merging/non-merging of entities at these subsequent lower levels, recursively following the same arithmetic rules of reticulation described (i.e., 1 + 1 = 1, 1 + 1 = 2, 1 + 1 = 3).

In scenario (1 + 1 = 2), the exchange of lower-level entities between two entities of the focal level will increase the average diversity and complexity at the focal level, while the diversity and complexity of the adjacent higher level remains unchanged; additionally, the average diversity and complexity at the adjacent lower level will also remain constant; and again, the trajectories of diversity and complexity at all subsequent lower levels will depend on the merging/non-merging of entities at these subsequent lower levels, recursively following the same arithmetic rules of reticulation.

In scenario (1 + 1 = 3), the reticulate formation of a novel entity at the focal level will both increase the average diversity and complexity at this level, but also the diversity and complexity of the adjacent higher level, while all lower-level average diversity and complexity values will remain unchanged; and, finally, the trajectories of diversity and complexity at all subsequent lower levels encompassed by the novel entity will depend on the merging/non-merging of entities at these subsequent lower levels, recursively following the same arithmetic rules of reticulation.

As a consequence, along with evolutionary divergence (ZFEL, [5]), evolutionary reticulation events will also increase the diversity and complexity in the system, if not at the focal level, then on the adjacent higher or lower levels. The increase in diversity and complexity of genes, macromolecules, organelles, cells, tissues, organs, individuals, groups, populations, species, clades, or higher-level units by reticulation is Biology’s Second Law.

### 3.4. The Role of Natural Selection, Other Forces, and Constraints

The two processes described above (divergence, reticulations) lead to the ongoing increase in diversity and complexity of entities at the different levels of the genealogical hierarchy. According to Eldredge [80] (p. 13), however, this ‘genealogical (informational or evolutionary) hierarchy represents transmission of heritable information, corresponding to the temporal dimension of life’, and is tightly connected with the ‘economic (ecological) hierarchy [that] represents dynamics of matter and energy exchange, and, generally, corresponds to the spatial dimension of life’. As a consequence, processes operating on the entities of the economic hierarchy (the ‘interactors’) leave their footprints in the entities of the genealogical hierarchy (the ‘replicators’). Natural selection and genetic drift, as two of these economically (ecologically) mediated processes, therefore, will act as forces that hold in check the forces responsible for the above-described ever-growing diversity and complexity of entities on the genealogical hierarchy side. Owing to the fact that this ‘dual hierarchical approach can be applied at levels above and below organism level’ [74] (p. 194), this model (the so-called ‘sloshing bucket’ model of Eldredge [81]) not only supports multi-level selection (MLS) theory but also adds important aspects to an evolutionary framework in which the waxing and waning of the diversity and complexity of biological entities is a consequence of a ‘balance of power’ between divergence and reticulation processes as diversity- and complexity-fostering forces and natural selection and genetic drift as their counterparts.

The rotational gain of importance of these two fundamental counter-balancing processes may also explain macroevolutionary phenomena like ‘punctuated equilibria’ [82,83], where bursts of increases in the diversity and complexity of evolutionary entities due to divergence and reticulations unchecked by selection alternate with periods of more balanced relationships between the two diverging processes. In a scenario of dramatic changes in environmental conditions, the ‘sloshing bucket’ model predicts that changes at higher levels of the economic (ecological) hierarchy (biosphere, biocenoses, ‘avatars’ = populations) will lead to corresponding changes among entities at and below the corresponding levels on the genealogical hierarchy side (species, populations = ‘demes’, individuals, genomes, genes). These changes on the genealogical hierarchy side will encompass on an enhanced level the processes of reticulate evolution (emergence of novel symbioses and endosymbioses, hybridisation among species, hybrid speciation events, enhanced gene flow among populations, merging of genomes, emergence of new gene combinations), creating higher levels of diversity and complexity (at least at lower hierarchical levels) as a starting point for evolutionary novelties and innovations.

### 3.5. Determinants for Trajectories of Reticulation

The theoretical considerations and empirical data presented above highlight the potential causes and consequences of reticulate evolution. This raises the following broader question: what factors generally determine the feasibility of evolutionary reticulations and influence the trajectories of entities following a reticulation event? Obviously, there is one decisive feature of evolutionary entities (regardless of their position in the genealogical and ecological hierarchy) that determines the outcome of their collision: the compatibility, reconcilability, or ‘mergerbility’ of entities. If a complete compatibility between two colliding entities is realised, a 1 + 1 = 1 scenario will result, while a partial compatibility will lead to a 1 + 1 = 2 and a complete incompatibility to a 1 + 1 = 3 scenario. As discussed earlier, the latter scenario fosters the emergence of a novel evolutionary entity, increasing diversity and complexity at the focal and the next-higher level. Therefore, in order to understand the patterns and boundary conditions of reticulate evolution, it is essential to identify the factors that determine the compatibility of entities at a given level of the evolutionary hierarchy.

As a first step towards generalising these underlying factors, we must recognise that the properties of entities at the focal level of a genealogical hierarchy are determined by (a) the characteristics and properties of entities at the next-lower level and (b) the emergent characteristics arising from the diversity of, and interactions among, entities at that next-lower level, recursively leading down to the lowermost level of genes and gene networks. In a multi-level feedback mechanism, therefore, lower-level entities set the boundary conditions for higher-level entities with regard to these higher-level entities’ ‘behaviour’ or ‘porosity’ in reticulation events, and are themselves the targets of entity collisions subsequent to a merger of entities at higher-level entities. As a consequence, research aiming at pinpointing the entity level responsible for upper-level entities’ behaviour in collisions will tremendously help in understanding the causes and consequences of reticulate evolution and will set the common theme of all research programmes dealing with evolutionary reticulations at all conceivable levels in the evolutionary hierarchy mentioned in the first part of the present review.

## 4. Towards a Research Programme Aiming at Causes and Consequences of Reticulate Evolution

Slightly paraphrasing Davies’ [84] notion provided in his book *The Goldilocks Enigma* dealing with the modern hypotheses of cosmology, that ‘life as we observe it today is 1 per cent physics and 99 per cent history’, evolution as we understand it today is also to an overwhelming extent a historical procedure governed by chance in the first place and to a minor degree by law-controlled necessity. However, in analogy to particle colliders in physics, which allow for the study of the internal structure of the components of matter, the collision of evolutionary entities provides insights into the very core of evolutionary mechanisms. With the appropriate theoretical background and suitable, powerful tools for analysing empirical data, this may allow evolutionary biologists to disentangle chance and necessity in evolutionary histories, move from a phenomenological–historical to a generalising–explanatory perspective, and flesh out the fundamental laws of evolutionary biology (if there are any) as a contribution to the initially mentioned underlying laws for evolving systems in general [14,16]. In the following, I would like to sketch the main research questions and tools necessary to approach these questions in order to engage in this endeavour pivotal to the biological sciences (see Table 1 for elements of the genealogical hierarchy, exemplary reticulate processes among them, and exemplary research topics on the causes, consequences, and boundary conditions of evolutionary reticulations, and Box A1 for examples of recent research findings in these fields).

### 4.1. Questions in the Study of Reticulate Evolution Under a Hierarchical Perspective of Evolution

*Which are the fundamental evolutionary entities in the genealogical and economical hierarchies?*—The presented hierarchical concept of evolution in general and reticulate evolution in particular rests on evolutionary entities that form vertices in hierarchically nested entity networks. Network science and network theory may therefore contribute the essential theoretical background for an innovative study of the evolutionary process in biology. As a consequence, it is essential in the first place to find out what these evolutionary entities at the different levels of the evolutionary hierarchy are and which entities in the genealogical hierarchy of the mentioned dual hierarchical model of biological systems proposed by Eldredge [80,81] correspond to which evolutionary entities in the economic (ecological) hierarchy.

*What is the nature of edges in network architectures of evolutionary entities?*—The relationships among entities at a focal level in the genealogical and economical hierarchies can be quite different. They can be uni- or bi-directional and neutral vs. promoting vs. inhibitory. The positions of entities in these networks can be central or marginal, dependent on the number and nature of the edges. These patterns among evolutionary entities may differ considerably between different hierarchical levels of the genealogical and economical hierarchies and also between the two hierarchy columns.

*What nature do the emergent forces that shape the upward directed ‘boundary conditions’ in the hierarchical dynamics of reticulate evolution have?*—The set of evolutionary entities at a given level of the two evolutionary hierarchies and the relationships among them will cause unpredictable, emergent properties at the adjacently next-higher levels. These properties will set the boundary conditions that determine the ‘evolutionary behaviour’ of these entities in the network of relationships at this upper level. They will also determine to which degree these emergent entities will be isolated from each other and to which degree the merging of or the exchange among these entities is possible.

*What determines the porosity of evolutionary entities?*—Reticulation between evolutionary entities is only possible when the borders between entities at a given level of the evolutionary hierarchies are not impermeable. Otherwise, the merger of two entities will only lead to the formation of a new entity at the adjacent next-higher level. This ‘porosity’ of evolutionary units is an emergent property resulting in the composition of and interactions among entities at the adjacent next-lower level, and its nature will be quite different at different evolutionary levels and in the two different hierarchy columns. Additionally, the reciprocal influence between corresponding levels in the two hierarchy systems will also contribute to the dynamics of this ‘porosity’.

*Are there Goldilocks Zones of reticulate evolution?*—Biological evolution appears to be a permanent alternation of the divergence (Biology’s First Law) and convergence (Biology’s Second Law) of entities at different levels of the evolutionary hierarchy. It seems that evolutionary entities hang in a balance between complete amalgation and fusion on the one hand and complete isolation and perpetual divergence on the other, and that this balance of opposite forces determines the fate of these entities in evolutionary history. The question arises whether there are ‘Goldilocks Zones’ for reticulation processes (too much would lead to complete fusion, too little would lead to complete isolation). What factors determine and shape these zones at the different levels of the evolutionary hierarchy? Are these factors and their strength constant throughout the evolutionary history or are there time slices along the geological scale in which ‘Goldilocks Zones’ of divergence/convergence processes shift and lead to punctuated equilibria?

### 4.2. Tools for Studying Reticulate Evolution at All Levels of the Evolutionary Hierarchy

*Machine learning tools*—Machine learning (ML) and other applications of artificial intelligence (AI) have rapidly emerged in molecular and evolutionary biology. From the perspective of research dealing with reticulate evolution, ML tools on the one hand support the extraction of characteristics from museum specimens for use in morphometrics or species and hybrid detection [87,88]. On the other hand, there are numerous applications of ML tools available nowadays in the field of molecular-based evolutionary biology, like the screening of genomes for introgressed loci (e.g., [89]), the analysis of gene expression patterns (e.g., [90]), the above-mentioned inference of historical introgression events (e.g., [91]), or the prediction of microRNAs, of promotor regions, and of transcription factor target genes [92]. All these methods may also be used in the different fields and levels of reticulate evolution research described in the following paragraphs.

*Network reconstruction tools*—When studying deep reticulate evolution with phylogenomic techniques, researchers are interested in the direction (donor vs. recipient lineage), the extent across the genome (genes vs. partial or whole genomes), the timing (in terms of geological epochs), and the mode (pulses of hybridisation vs. continuous gene flow) of introgressive hybridisation [93]. To address these questions, the last 15 years have seen tremendous activities in the development of software programmes and analytical pipelines placed at the disposal of evolutionary biologists to detect, analyse, and pinpoint genetic and genomic introgression events [89,91,93,94] and homoploid or polyploid hybrid speciation events [95,96,97,98]. This active field of phylogenomics will gain an even broader interest than at present by realising the paramount importance and the pervasive presence of reticulations in eukaryote evolution.

*Analysis of transcriptomes and genetic regulatory circuits in hybrids*—Newly formed hybrids and homoploid or polyploid hybrid lineages have been shown to gain innovative potential due to changes in gene expression patterns and the formation of novel genetic regulatory circuits (‘hybridisation shock’). The observation of shared transcriptional patterns after hybridisation and polyploidisation across the three kingdoms of plants, animals, and fungi [99] points towards comparable evolutionary mechanisms acting at the genetic and genomic level that could be generalised for all eukaryotes. Despite a half-century long history of hybrid gene expression research, there are still many outstanding questions in this field addressing the role of gene expression in hybrid fertility, development, and stability, along with the ecological adaptation of hybrid lineages [100]. Software programmes like the recently developed HYBRIDEXPRESS [101] may contribute significantly to these analyses of the complex, non-additive effects at the transcriptional level of hybrids and allopolyploids. Additionally, intensified research on homoploid hybrid speciation and the connected transcriptional changes and ecological adaptations will further broaden the empirical basis for this still underestimated speciation mode (e.g., [102]).

*Analysis of epigenetic consequences of hybridisation*—As transcriptional activities are also influenced by DNA methylation, histone modifications, and non-coding regulatory RNA pathways (e.g., miRNAs, lngRNAs), these epigenetic modifications play a pivotal role during the development of multicellular organisms [103]. Hybridisation leads to a remodelling of the epigenetic landscape (‘hybridisation shock’) that may have both detrimental but also positive and evolutionary innovative consequences. While early-generation hybrids may suffer from misexpression due to the break-up of interaction cis/trans regulatory elements, changes in the regulation networks of small RNAs, and the burst of transposable elements [100,104], these dramatic changes are also providing the necessary developmental, morphological, physiological, and ecological innovations for adaptational saltations and novel evolutionary lineages (‘hopeful monsters’, [105,106,107,108]; ‘cataclysmic evolution’, [109]). Additionally, owing to the important function of epigenetic modifications for the preservation of genomic integrity by keeping transposable elements in check, epigenetic changes caused by hybridisation also play a role in chromosomal rearrangements and hence the genome architecture of homoploid and polyploid hybrid lineages [110,111].

*Analysis of chromosomal architecture in hybrids and polyploids*—The research on evolutionary reticulations both in shallow and deep phylogenetics has tremendously benefitted from progresses in cytogenetics and cytogenomics because these methods (karyotype analysis, chromosome banding, FISH, GISH) enable evolutionary scientists to study the above-mentioned large-scale changes in the chromosomal architecture of hybrid lineages [52]. Often used in conjunction with phylogenetic reconstructions nowadays, modern cytogenetic and cytogenomic techniques (‘chromosome painting’) allow both the reconstruction of reticulate speciation events (homoploid and polyploid hybrid species formations) in recent evolutionary times (e.g., [112,113]) and the tracking of changes in genome architecture as a consequence of these reticulations further back in time (e.g., diploidisation; [114,115,116]). Progress in whole-genome sequencing and whole-genome assembly methods is complementing chromosome painting approaches [117] and will provide micro- and macrosyntenic information for more precise phylogenetic network reconstructions [51].

*Modelling determinants and boundary conditions of reticulate evolution*—A final field of methodological innovation in the research on reticulate evolution is here identified in mathematical modelling and computer-aided simulation approaches to hybridisation. Again, closely connected with questions addressing the build-up of reproductive isolation in speciation (e.g., [70]), the modelling of hybridisation (e.g., [69,118]) allows for predictions about the role and interplay of the extrinsic (environmental) and intrinsic (genetic, epigenetic, chromosome architectural) circumstances giving rise to hybridisation, controlling its frequency, and shaping its evolutionary outcomes. Additionally, even chromosomal rearrangements after hybridisation events could be modelled [119] and compared with the empirical findings of these changes in the genome architecture and their consequences for the formation of reproductive isolation between lineages [120,121].

## 5. Conclusions

Evolution is reticulate. Reticulation increases diversity and complexity—if not at the focal level, then on adjacent lower and/or upper levels in the evolutionary hierarchy. Reticulation processes, therefore, act—in addition to the tendency for diversity and complexity to increase in unchecked evolutionary systems by ongoing divergence (‘Zero-Force Evolutionary Law’, ‘Biology’s First Law’; [5])—as a second mechanism for the establishment of evolutionary novelty and the rise in biodiversity and biocomplexity (‘Biology’s Second Law’), providing the raw material for subsequent diversity-confining drift and selection processes. In order to fully appreciate reticulation processes as part of an updated paradigm of evolutionary biology, a research programme on the topic should encompass (a) the identification of the fundamental evolutionary entities as vertices and (b) the study of the relationships among these vertices as edges in the resulting network architectures, along with surveys on the underlying determinants, leading to (c) emergent boundary conditions for reticulations and (d) the porosity of evolutionary entities, and should finally (e) address the question whether there are equilibrium conditions between the complete fusion and complete isolation of evolutionary entities (‘Goldilocks Zones’) that foster reticulate evolution. As tools in this research programme, machine learning and modelling approaches, along with methods in the field of network reconstruction, transcriptomics, epigenetics, and karyology, are identified.

## Figures and Tables

**Table 1:** Elements of the genealogical hierarchy (vertices of upper-level evolutionary networks) and exemplary reticulate processes among them (edges of upper-level evolutionary networks), along with possible research topics on causes, consequences, and boundary conditions of evolutionary reticulations.

**Elements of genealogical hierarchy (vertices)**	**Exemplary reticulate processes among elements (edges)**		**Exemplary research topics on causes (a), consequences (b), and** **boundary conditions (c) of evolutionary reticulations**
**Genes, non-coding elements, mobile genetic elements**	**1 + 1 = 1:**	Mobile DNA element incorporation, gene fusion	**a:** Causes for activation of mobile elements **a:** Causes for non-homologous crossing-overs **a:** Causes for exonisations and intronisations **b:** Evolution of novel gene functions or alternative spicing patterns **b:** Evolution of novel regulatory circuits **b:** Non-functionalisation, pseudogenisation **b:** Gene fusion **c:** Control mechanisms of mobile element activation **c:** Influence of epigenetic changes **c:** Control mechanisms of recombination processes
	**1 + 1 = 2:**	Recombination, domain exchange, exon-shuffling	
	**1 + 1 = 3:**	Domain accretion, exon fusion, locus interactions	
**Chromosomes**	**1 + 1 = 1:**	Chromosome fusion	**a:** Chromosome breakpoints **a:** Factors affecting chromosomal translocations **a:** Holocentric chromosomes **b:** Karyotype evolution **b:** Alternation of tandem repetitive DNA arrays and centromeres **b:** Changes in basic chromosome numbers **b:** Changes in gene linkage and recombination rates **c:** The role of genes, non-coding elements, mobile DNA elements in chromosome structure and functioning **c:** The role of epigenetic modifications in structural changes in chromosomes **c:** The role of genome architecture for chromosomal behaviour
	**1 + 1 = 2:**	Homologous (reciprocal) translocations	
	**1 + 1 = 3:**	Novel chromosome formation by duplication and non-homologous translocations	
**Genomes, extra-nuclear genomes**	**1 + 1 = 1:**	Genome fusion, uni-directional intergenomic DNA transfer	**a:** The role of mobile elements in intergenomic DNA transfer **a:** The mechanism of chromosomal crossing-overs and chiasmata formation **a:** Meiotic aberrations leading to non-disjunctions and formation of unreduced gametes **b:** Formation of novel gene regulation networks **b:** Stoichiometry of gene products encoded on different genomes **b:** Nucleotypic effects **c:** Effectiveness of intergenomic communication and conflict **c:** The role of mitochondrial and plastidic fusion processes **c:** The role of karyotypes in genome (in)compatibilities
	**1 + 1 = 2:**	Bi-directional intergenomic DNA transfer	
	**1 + 1 = 3:**	Polyploidy	
**Cells, organelles**	**1 + 1 = 1:**	Cell or protoplast fusion (e.g., zygote or endosperm formation)	**a:** Reproductive cell fusions in sexual eukaryotes **a:** Cell–cell fusion in slime moulds **a:** Dikaryon formation in fungi **a:** Cell engulfment **b:** Syncytia formation **b:** Consequences in cancer (tumour progression, drug resistance, tumour diversity and heterogeinity, immune evasion) **b:** Gain of new physiological functions (e.g., for nutrient and gas exchange), tissue regeneration, natural defence against cancer **b:** Transfer of learned behaviour via cell fusion (cf. [85]) **b:** Compartmentalisation of reaction systems **c:** Extracellular signals triggering differentiation and fusion competence **c:** Cellular, molecular, and biophysical factors determining cell–cell recognition and tight adhesion **c:** Factors leading to phagocytosis avoidance or to resistance against killing after ingestion
	**1 + 1 = 2:**	Horizontal DNA transfer	
	**1 + 1 = 3:**	Endosymbiosis	
**Organisms, individuals**	**1 + 1 = 1:**	Chimaera formation	**a:** Somatic fusion between multicellular individuals (cf. [86]) **a:** Nutritional, communicational, physiological, and social causes of uni- and bi-directional cell transfer **a:** Causes of sexual propagation **b:** Fitness consequences through complementation (chimeric vigour) **b:** Transfer of immunity **b:** Negative genetic, developmental, or physiological effects of sexual propagation (lack of uniformity, longer juvenile period, disease vulnerability, fitness reduction) **b:** Positive effects of sexual propagation (genetic diversity, hybrid vigour, heterosis effects, adaptability) **c:** Evolution of allorecognition **c:** Evolution of eusociality **c:** Evolution of sexuality, advantages and disadvantages of sex
	**1 + 1 = 2:**	Trophallaxis, vertically transferred maternal immune cells	
	**1 + 1 = 3:**	Sexual propagation	
**Populations, subspecies, demes, ecotypes**	**1 + 1 = 1:**	Fusion in metapopulation systems	**a:** Climatic, geomorphological, and geological changes promoting barrier breakdown **a:** Intrinsic and extrinsic factors promoting dispersal processes **a:** Factors promoting contact between divergent populations of a species and establishment of founder populations **b:** Loss of differentiation among populations or population groups **b:** Formation of migration–selection tension zones (primary hybrid zones) **b:** positive (adaptability) or negative (outbreeding depression) consequences of gene flow among populations of a species **c:** Factors determining the fate of primary and secondary hybrid zones **c:** Factors promoting establishment of ecologically novel founder populations (e.g., habitat suitability, niche availability or formation, invasiveness of novel ecotypes)
	**1 + 1 = 2:**	Migration of individuals/diaspores among populations	
	**1 + 1 = 3:**	Formation of novel (founder) populations from mergers of different (morphologically, physiologically, or ecologically divergent) demes of a species	
**Species**	**1 + 1 = 1:**	Merging of species	**a:** Intrinsic and extrinsic determinants of hybrid formation, viability, and fitness **a:** Factors influencing backcrossing and the balance of gene flow and selection in a hybrid zone **a:** Architectures of reproductive isolation **b:** Species loss through hybridisation **b:** The role of adaptive hybridisation **b:** Deep reticulations in phylogeny **c:** Hybrid zone types and their dynamics **c:** Factors of reproductive isolation of homoploid hybrid lineages **c:** Intrinsic and extrinsic determinants of allopolyploid establishment **c:** Reinforcement, evolution of prezygotic barriers
	**1 + 1 = 2:**	Introgressive hybridisation	
	**1 + 1 = 3:**	Hybrid speciation (homoploid, polyploid)	
**Clades**	**1 + 1 = 3:**	Holobiont assembly, ecosystem assembly, symbiosis (mutualism, parasitism, commensalism), holobiont formation, endosymbiosis	**a:** Mechanisms of holobiont assembly **a:** Mechanisms of ecosystem assembly **a:** Mechanisms of symbiosis establishment **b:** Benefits (enhanced resilience, expanded metabolic capabilities, enhanced adaptability) and risks (dysbiosis and disease, dysregulation of immune systems, vulnerability to stressors, complex genetic flux caused by viruses) of holobiont formation **b:** Enhanced or reduced stability of ecosystems and resilience against abiotic and biotic changes **b:** Morphological, physiological, evolutionary, and ecological consequences of symbiosis (e.g., novel life-forms, new capabilities, accelerated speciation, evolution of keystone organisms) **c:** Intrinsic and extrinsic determinants of species compatibility **c:** Factors determining ecosystem assembly and stability **c:** Determinants of shifts in symbiotic behaviour

## Data Availability

No new data were created or analysed in this study. Data sharing is not applicable to this article.

## Appendix A

**Box** **A1** Examples for studies aiming at causes and consequences, up- and downward causations, or boundary conditions (‘Goldilock Zones’) of evolutionary reticulations at the different levels of the biological hierarchy as specified in Table 1.  *Genes, non-coding elements, mobile genetic elements*—Besides recombination, reticulate evolutionary processes at the DNA sequence level in eukaryotes are becoming more and more associated with the activity of parasitic repetitive DNA, called transposable elements (TEs). Betancourt and colleagues [122] provide an updated overview on the causes and consequences of TE activity from an evolutionary perspective, give evolutionary and ecological explanations for the variation in TE activity based on their beneficial and harmful effects, and list evolutionary adaptations directly or indirectly mediated by TE activity on higher levels of the biological hierarchy (e.g., chromosome organisation, genome architecture and size, [123]), along with more proximal effects like gene function or gene regulation (e.g., activation of anthocyanin biosynthesis in *Capsicum annuum,* [124]). For example, a rapid increase in genome size as a consequence of TE hyperactivity has been recently reported for a species of the wood-white butterfly genus *Leptidea* [125] and parallels the exceptional intraspecific variability in chromosome numbers (2*n* = 56–106) found in this species *(L. sinapis)* in its large Eurasian distribution range [126]. The accumulation of TEs observed in natural killifish hybrids between two members of the genus *Fundulus* [127], on the other hand, exemplifies a downward causation from a reticulation event at a higher level of the biological hierarchy (species) on this lowermost level. The detrimental effects of excessive TE accumulation observed in annual African killifish in the course of adaptation to seasonal habitat desiccation [128] and ageing [129], on the one hand, and the beneficial effects of TE activity in terms of fostering higher levels of genetic variation as a prerequisite for evolutionary adaptation (e.g., in *Capsella* [130]) or of facilitating the formation of de novo genes (with many examples given in [131]), on the other, argue for the presence of ‘Goldilock Zones’ for TE activity.  *Chromosomes*—The organisation of the eukaryotic genome into chromosomes (i.e., linkage groups of individual genes) is tightly linked to meiotic recombination as a fundamental feature of sexually reproducing species, for which its genetic basis was recently reviewed by Johnston [132]. The frequency and outcome of meiotic recombination in turn is considerably influenced by structural chromosomal features resulting from a broad array of mechanisms at the DNA level [133]. Therefore, examples of upward causations influencing evolutionary reticulation at the chromosomal level (e.g., recombination, chromosomal rearrangements) are genes directing the frequency, the locality, and the consequences of DNA double-strand breaks [133], or TE activities leading to novel or changed satellite DNA (satDNA) formations in centromeres and telomeres [134,135]. In terms of upward causations of evolutionary reticulations at the chromosomal level, a recent review by Wang and colleagues [136] summarises how genome architecture impacts the evolution of reproductive barriers and potentially speciation rates in plants and animals. Recent examples of studies aiming at karyotype diversification and chromosome rearrangements in squamate reptiles [137] or bees [138], along with those aiming at the role of TE in genome evolution studied through the whole-genome sequencing of closely related species (e.g., *Brassica,* [139]; *Biscutella,* [140]), represent macroevolutionary (phylogenetic) and microevolutionary (mechanistic) approaches to genome architectural questions. The extremely complex machinery of proteins involved in the control and repair of meiotic DNA double-strand breaks (the ‘recombinosome’, see [141]), the observed changes in recombination rates (e.g., leading to DNA deletion called ‘genome downsizing’ after whole-genome duplications in plants; [142]), and the observation of centromere diversity (centromere position, number, distribution, or strength) having considerable evolutionary impacts on karyotypes and reproduction [143] argues for ‘Goldilock Zones’ between too few and too many reticulation processes also on this level of the evolutionary hierarchy.  *Genomes, extra-nuclear genomes*—In eukaryotes, evolutionary reticulations between different genomes are caused either by a combination of nuclear genomes after homo- or polyploid hybridisation or by uni- or bi-directional intergenomic DNA transfer between nuclear, mitochondrial, and/or plastidic genomes. Again, the role of mobile DNA elements (at the DNA level) or of the factors determining the frequency and location of recombination processes (at the chromosomal level) constitute important upward-directed boundary conditions for these genomic reticulations, while factors caused by cell engulfment processes, the fusion of cells or organisms, and hybridisation between species or demes, along with genomic exchanges among members of holobionts or symbioses, represent examples of downward-directed conditions determining the feasibility and the outcome of genomic exchanges. A phylogenetic approach spanning 1 billion years of plant evolutionary transitions from algae to angiosperms revealed the remodelling of mitochondrial and plastidic genomes, which has led to organelle proteomes adapted to macroevolutionary trends, especially for the settling of land [144]. The transfer of genes from the plastidic and/or mitochondrial genome to the nuclear genome has happened in a tremendous expanse during organelle evolution and is continuously ongoing at the present [145,146]. An extremely important consequence of these gene transfers is the necessity of keeping stoichiometric balances among gene products coded on different genomes but being members of a joint regulatory or metabolic network or even being parts of a functional protein complex (e.g., ribulose-1,5-bisphosphate-carboxylase/-oxygenase with the genes *rbcL* coded on the chloroplast and *rbcS* on the nuclear genome or members of the mitochondrial oxidative phosphorylation complexes). These gene regulatory challenges are at the focus of many studies in the animal and plant kingdom (e.g., [147,148]), but have gained special attention in conjunction with polyploidy in plants [149,150,151,152,153].  *Cells, organelles*—Reproductive cell–cell fusion is an extremely common process in sexual eukaryotes, which arose very early in the evolution of this organism group, and consists of the species-specific attachment of gametic membranes and the fusion of their lipid bilayers, the molecular mechanisms, however, remaining not fully understood [154]. In addition to its paramount role in reproductive biology, plasmogamy has also been observed in unicellular eukaryotes in reaction to stress under unfavourable environmental conditions [155,156], and in the formation of syncytia (or multinucleate ‘coenocysts’) in fungi [157] and slime moulds (‘plasmodial fusion’, [158]). In the unicellular slime mould *Physarum*, cell–cell fusions enabled the transfer of a learned response to a repellent and raised speculations that similar phenomena may occur in other cell–cell fusion systems [85]. Besides the complete fusion of cells, the intercellular exchange of cell organelles is a further evolutionary reticulation process on this level of the biological hierarchy. Valenti and colleagues discuss the biological relevance, mode, and mechanisms underlying the intercellular trafficking of mitochondria in mammalian cells, which was observed both in physiological and pathological conditions [159], while kleptoplasty (sequestration of chloroplasts from algal food in the intestinal cells of some sea slugs) even involves members of two different phyla [160].  *Organisms, individuals*—The mergers of complete organisms seem to be a rare process in multicellular eukaryotes, while they are more common in multinucleate, unicellular organisms like the above-mentioned slime moulds [161] or as the result of human intention (‘plant grafting’, [162]). However, in a recent review, Rinkevich and Goulet provide evidence that chimerism (i.e., the presence of cells with different genetic compositions within a single organism) is widespread in nature and can be observed in more than ten phyla of protists, plants, invertebrates, and vertebrates as a result of a fusion of conspecifics or the incorporation of cells from different individuals [163]. Marine invertebrates especially seem to be an animal group in which somatic chimerism is common and established either between young/laval partners (sponges, corals, echinoderms) or even between juveniles or adults (tunicates). As a consequence, chimaera-forming tunicates are important study systems for the evolution and molecular mechanisms of allorecognition [164,165,166], but also fungi are studied in this respect [86,167]. Since somatic chimerism could be mutually beneficial, but is also associated with costs (often depending on the relatedness of fusing individuals) [86], up- and downward-directed factors causing chimaera formation and the determination of a possible ‘Goldilocks Zone’ on this level of the biological hierarchy may be interesting fields for future research.  *Populations, subspecies, demes, ecotypes*—The causes and consequences of reticulate processes among populations as groups of individuals within a metapopulation system fall into the realm of ecological research and are usually studied with population genetic or phylogeographical tools under the consideration of many biotic and abiotic factors. Here, functional features determining the potential and realised ecological niche of a species or species subgroup, the potential and intensity of dispersal to other locations, and the potential to invade conspecific populations play important roles in forming boundary conditions for reticulations at this level of the biological hierarchy. Examples exist, especially in animal species (e.g., in giraffe metapopulations, [168]), where subpopulations overlap in space, but remain isolated from each other not due to environmental but to social factors. Additional complexity concerning questions of the causes, consequences, and boundary conditions of metapopulation reticulations emerges when populations show ecological divergence and genetically (and often morphologically) different demes of a species collide or exchange members (see a recent review of the ecotype concept by Johannesson and colleagues [169]). In many of these cases, equilibrium conditions emerge in which gene flow across ecotype boundaries is countered by different selection regimes in the sink populations preferring the local ecotype over the foreign one, leading to more-or-less steep transitions in the contact zone. Since these conditions could be the starting point for the formation of a species with the establishment and enforcement of pre- and post-mating isolation mechanisms, there is an extremely broad literature base available on the genetic, chromosomal, and genomic upward-directed causations determining the evolutionary trajectories of ecotypes. Conversely, only few examples exist where hybridisation between two ecotypes leads to novel lineages independent of their precursors. Curran and colleagues [170] report on an African grass species *(Allopteris angusta)* in which hybridisation facilitates the geographical dispersal of both ecotypes involved by the enlargement of their ecological niches via novel gene (and trait) combinations.  *Species*—Owing to the paramount importance of the species level in research on biodiversity and evolution, there is an ample foundation of research results focussing on the causes and consequences of species collisions (e.g., [8,9,11,171]). Possible outcomes of these collisions range from the complete merging of the two species involved (‘extinction by hybridisation’), over the transfer of morphological, physiological, or ecological characteristics in one or both directions (introgressive hybridisation), to the formation of novel and independently evolving hybrid lineages (i.e., species). The latter process especially, either in conjunction with genome duplication (allopolyploid hybrid speciation) or not (homoploid hybrid speciation), is responsible for the deep phylogenetic reticulations seen in whole-genome sequencing studies both in animals and plants [42,43]. Owing to the (in most cases) long-lasting processes triggered by hybridisation between species (paralleling the long-lasting processes of speciation; the ‘speciation continuum’ [172]), research aiming at factors determining the causes and consequences of species collisions is usually indirect and follows either ‘magnifying glass’ [architecture of (incomplete) reproductive isolation] or ‘spyglass’ approaches (deep reticulations in phylogeny). As a consequence, generalisations about boundary conditions determining the occurrence and the outcome of hybridisation and questions aiming at ‘Goldilocks Zones’ for hybrid speciation are more and more approached through mathematical modelling (e.g., [56,69]), providing hypotheses that could be tested subsequently with empirical data or experiments.  *Clades*—In contrast to the species level discussed above, the complete merging of entities above the species level (clades) or the exchange of lower-rank evolutionary units (species) between clades is not observed in nature owing to the abstract nature of the phylogenetic hierarchy. What is realised in tremendous diversity and complexity, however, is the temporal or permanent fusion of species from different clades of the Tree of Life in biotic assemblages evolving conjointly as homogenous units and independently from their original lineages. The resulting species-networks can range between loosely organised (ecosystem communities, holobionts) and tightly linked and obligate assemblages (symbioses, endosymbioses). In terms of the causes, consequences, and boundary conditions of reticulations at this level of the biological hierarchy, extremely interesting studies and experimental approaches are followed: Harrower and Gilbert [173] studied the parasitism–mutualism continuum in the system of *Yucca brevifolia* and arbuscular mycorrhiza fungi along a climatic gradient and found in an experiment that fungal community structures with an initially negative impact on *Yucca* fitness turned positive after several months. Kamal and colleagues [174] were able to observe the incipient stages of endosymbiosis between the ciliate *Tetrahymena utriculariae* and its algal partner *Micractinium tetrahymenae* and demonstrated that mitochondrial remodelling and endosymbiont metabolic reprogramming regulate the transitions between mutualistic and parasitic stages in dependence of environmental conditions (oxygen presence/absence). In relation to assembly processes leading to ecosystem communities, Leibold and colleagues provided evidence that community ecology ignoring evolutionary processes like adaptation misses an important factor that governs boundary conditions of this fundamental process of community ecology and biodiversity research [175].
